# A Multimodal Risk Network Predicts Executive Function Trajectories in Non-demented Aging

**DOI:** 10.3389/fnagi.2021.621023

**Published:** 2021-09-16

**Authors:** Shraddha Sapkota, G. Peggy McFall, Mario Masellis, Roger A. Dixon

**Affiliations:** ^1^Hurvitz Brain Sciences Research Program, Sunnybrook Research Institute, Sunnybrook Health Sciences Centre, Toronto, ON, Canada; ^2^Department of Psychology, University of Alberta, Edmonton, AB, Canada; ^3^Neuroscience and Mental Health Institute, University of Alberta, Edmonton, AB, Canada; ^4^Department of Medicine (Neurology), University of Toronto, Toronto, ON, Canada

**Keywords:** modifiable risk factors, genetic risk scores, normal aging, cognitive trajectories, Alzheimer’s disease, Victoria Longitudinal Study

## Abstract

**Background:** Multiple modalities of Alzheimer’s disease (AD) risk factors may operate through interacting networks to predict differential cognitive trajectories in asymptomatic aging. We test such a network in a series of three analytic steps. First, we test independent associations between three risk scores (functional-health, lifestyle-reserve, and a combined multimodal risk score) and cognitive [executive function (EF)] trajectories. Second, we test whether all three associations are moderated by the most penetrant AD genetic risk [*Apolipoprotein E* (*APOE*) ε4+ allele]. Third, we test whether a non-*APOE* AD genetic risk score further moderates these *APOE* × multimodal risk score associations.

**Methods:** We assembled a longitudinal data set (spanning a 40-year band of aging, 53–95 years) with non-demented older adults (baseline *n* = 602; *M*age = 70.63(8.70) years; 66% female) from the Victoria Longitudinal Study (VLS). The measures included for each modifiable risk score were: (1) functional-health [pulse pressure (PP), grip strength, and body mass index], (2) lifestyle-reserve (physical, social, cognitive-integrative, cognitive-novel activities, and education), and (3) the combination of functional-health and lifestyle-reserve risk scores. Two AD genetic risk markers included (1) *APOE* and (2) a combined AD-genetic risk score (AD-GRS) comprised of three single nucleotide polymorphisms (SNPs; *Clusterin*[rs11136000], C*omplement receptor 1*[rs6656401], *Phosphatidylinositol binding clathrin assembly protein*[rs3851179]). The analytics included confirmatory factor analysis (CFA), longitudinal invariance testing, and latent growth curve modeling. Structural path analyses were deployed to test and compare prediction models for EF performance and change.

**Results:** First, separate analyses showed that higher functional-health risk scores, lifestyle-reserve risk scores, and the combined score, predicted poorer EF performance and steeper decline. Second, *APOE* and AD-GRS moderated the association between functional-health risk score and the combined risk score, on EF performance and change. Specifically, only older adults in the *APOE*ε4− group showed steeper EF decline with high risk scores on both functional-health and combined risk score. Both associations were further magnified for adults with high AD-GRS.

**Conclusion:** The present multimodal AD risk network approach incorporated both modifiable and genetic risk scores to predict EF trajectories. The results add an additional degree of precision to risk profile calculations for asymptomatic aging populations.

## Introduction

Worldwide projections show expected increases in the prevalence of dementia (Prince et al., 2015; Livingston et al., 2017; Mapstone et al., 2017; Wimo et al., 2017). It is estimated that by delaying the onset of Alzheimer’s disease (AD) by 5 years, prevalence may be reduced by ∼50% (Reitz, 2016). Epidemiological evidence points toward a multifactorial etiology for cognitive changes in aging (Wang et al., 2019). A diverse set of risk and protective factors that co-occur over the lifespan have shown differential and dynamic influence on cognitive trajectories in aging (Anstey et al., 2014; Roberts et al., 2015; Zahodne et al., 2016; Van der Linden and Juillerat Van der Linden, 2018; Gilsanz et al., 2019; Wang et al., 2019). Complex multimodal networks (Badhwar et al., 2020) of non-modifiable (genetic) and modifiable risk factors (i.e., interactions and risk indexes; Dixon and Lachman, 2019) may identify asymptomatic older adults with high risk profiles and provide opportunity to delay accelerated cognitive decline.

Some recent studies have adopted a multimodal risk score approach whereby a composite of AD risk exposure is obtained by summing across risk indicators representing different risk domains (Hooshmand et al., 2018; Andrews et al., 2019; Deckers et al., 2020). There are several advantages to a risk score approach over single risk indicators, including (1) the incorporation of multiple risk factors into a single score (Hall et al., 2003), (2) relatively consistent risk scores across populations (D’Agostino et al., 2001), and (3) the concept of a high or low risk score can easily be explained to the general public (Reitz et al., 2010). Such risk scores have been developed with both modifiable and genetic risk factors. Some previously developed risk scores include the (1) the LIBRA index (Deckers et al., 2017; Vos et al., 2017; Schiepers et al., 2018) using only modifiable risk indicators to predict different cognitive domains (processing speed and memory) in a range of age groups (e.g., midlife versus oldest old), (2) the Cardiovascular Risk Factors, Aging and Dementia Study (CAIDE) Dementia Risk Score (Stephen et al., 2017) included age, gender, obesity, hyperlipidemia, hypertension, physical inactivity, and *Apolipoprotein E* (*APOE*) ε4 carrier status as risk indicators to estimate dementia risk (Enache et al., 2016) and cognitive profile of healthy adults (Ecay-Torres et al., 2018), (3) an AD polygenic risk score and AD status to predict clinical diagnosis (e.g., AD, vascular and mixed dementias) (Stocker et al., 2020), (4) the Australian National University-AD Risk Index (ANU-ADRI) is a validated (Anstey et al., 2014) self-report index measure on 15 risk factors (i.e., age, education, depressive symptoms, and physical inactivity) to predict dementia incidence (Anstey et al., 2013), and (5) a summary risk score developed to predict AD in older adults using vascular risk indicators and the *APOE* ε4+ allelic risk (Reitz et al., 2010). Genetic risk scores (Stocker et al., 2018) in non-demented older adults have been developed with polygenic risk scores (Andrews et al., 2016) and candidate gene single nucleotide polymorphisms (SNPs; Sapkota and Dixon, 2018). For example, in our previous work (Sapkota et al., 2015, 2017; Sapkota and Dixon, 2018), we established an AD-genetic risk score (AD-GRS; *Clusterin* [*CLU*; rs11136000] + C*omplement receptor 1* [*CR1*; rs6656401] + *Phosphatidylinositol binding clathrin assembly protein* [*PICALM*; rs3851179]) and a cognitive aging genetic risk score that influences executive function (EF) performance and change as modified by *APOE* (rs7412, rs4293580) in non-demented older adults. Notably, high AD-GRS risk magnified the risk associated with increasing cognitive aging genetic risk selectively for *APOE* ε4+ carriers on EF performance.

**FIGURE 1:**
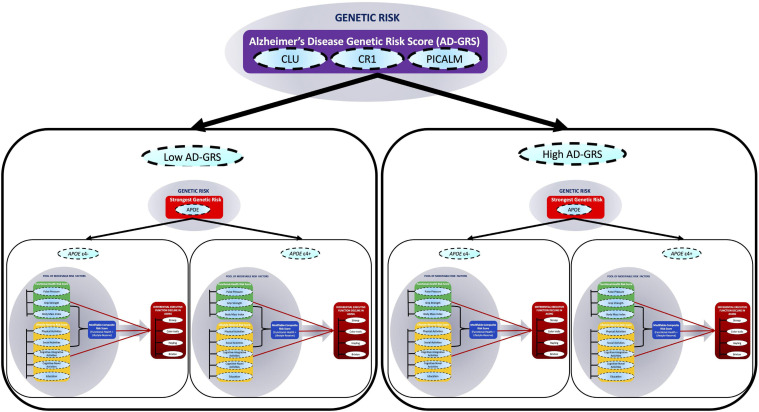
A multimodal risk network predicts executive function (EF) trajectories in non-demented aging. In this network, we include (1) a pool of eight modifiable risk factors [pulse pressure, grip strength, body mass index, physical activities, social activity, cognitive-integrative activity, cognitive-novel activity, and education], (2) four single nucleotide polymorphisms (SNPs) (*APOE*, *CLU*, *CR1*, and *PICALM*), and (3) four standard cognitive tests (Stroop, Color trails, Hayling, and Brixton). The modifiable risk factors are clustered into two main modifiable domain risk scores (functional-health and lifestyle-reserve) and a Modifiable-Composite Risk Score (M-CRS; functional-health + lifestyle-reserve). Functional-health risk score ranges from 0 to 5, lifestyle-reserve risk score ranges from 0 to 6, and the M-CRS ranges from 0 to 11. The SNPs are represented as a key AD genetic risk (*APOE*) and an Alzheimer’s disease genetic risk score (AD-GRS; *CLU* + *CR1* + *PICALM*). The cognitive tests are combined to represent EF in aging. Within this network, first, we examine whether the three modifiable domain risk scores (functional-health, lifestyle-reserve, and a M-CRS) predict differential EF decline in aging. Second, we test whether each of the three predictions are moderated (1) by a key AD genetic risk factor (stratified into *APOE* ε4– versus ε4+) and (2) further moderated by an AD-GRS (as stratified into low and high AD-GRS), to predict differential EF performance and decline in non-demented aging.

In the present study, we extend this work by using longitudinal data to test a multimodal risk network that integrates both modifiable and genetic risk factors to predict differential cognitive trajectories in non-demented aging (Medina et al., 2017). Specifically, we test whether associations between three modifiable risk scores and EF trajectories are moderated by key AD genetic risk scores. We emphasize that we are not predicting AD risk but using the AD risk factors to predict cognitive decline in non-demented aging. We use the term “network” to refer to the combination of modifiable and genetic risk domains represented with multiple indicators within each domain. Our multidomain network is represented and tested as follows. First, a pool of eight modifiable risk factors are clustered into two main modifiable domain risk scores {functional-health [pulse pressure (PP) + grip strength + body mass index (BMI)], lifestyle-reserve [physical activities + social activities, cognitive-integrative information processing + cognitive-novel information processing + education]} and a Modifiable-Composite Risk Score (M-CRS) combining functional-health + lifestyle-reserve risk score. Second, four SNPs (*APOE*, *CLU*, *CR1*, and *PICALM*) are represented as a key AD genetic risk (*APOE*) and an AD-GRS (*CLU* + *CR1* + *PICALM*). Third, within this network, we examine whether the three modifiable domain risk scores predict differential EF performance and decline. Fourth, we test whether each of the three predictions are moderated (1) by a key AD genetic risk factor (stratified into *APOE* ε4− versus ε4+) and (2) further moderated by an AD-GRS (as stratified into low and high AD-GRS), to predict differential EF performance and decline in non-demented aging (see Figure 1). This study provides several novel contributions toward cognitive aging risk score predictions. First, we incorporate both modifiable risk scores and genetic risk scores, to predict EF trajectories. Second, we use an accelerated longitudinal design to test how risk scores predict EF trajectories across a unique 40-year band of aging in non-demented older adults. Third, we test whether a complex network of multimodal risk scores predicts EF. Specifically, whether genetic risk scores moderate modifiable risk scores to predict EF.

The most prominent and consistently associated genetic risk factor for AD and cognitive decline is the *APOE* (chromosome 19q13.2) ε4 allele (Liu et al., 2013). *APOE* ε4 allelic risk is associated with increased risk for sporadic AD and accelerated cognitive decline whereas, the ε2 and ε3 alleles are considered to have protective and neutral effects, respectively (Liu et al., 2013). Non-demented older adults with *APOE* ε4 risk have higher amyloid pathology, medial temporal lobe atrophy, neuroinflammation, and ε4/ε4 homozygotes show the highest cerebral amyloid angiopathy (Nelson et al., 2013). A recent study using community-dwelling older adults showed that ε4 carriers had faster EF and verbal fluency decline than ε2 carriers and ε3 homozygotes (Reas et al., 2019). Inconsistent findings for *APOE* genetic risk versus polygenic risk score (PGRS) with *APOE* have also been reported. For example, a recent study showed that non-demented older adults with high PGRS are associated with cognitive decline but this association was no longer present when *APOE* genotype was removed (Porter et al., 2018). *APOE* ε4 allelic risk has also been linked to steeper memory decline in normal aging and cognitive decline in MCI (Albrecht et al., 2015). In addition to *APOE*, genome wide association studies (GWAS) have linked several other SNPs to increased dementia susceptibility, including: rs11136000 located within an intron of *CLU* on chromosome 8, rs6656401 at position 207692049 located near *CR1* on chromosome 1, and rs3851179 is located 88.5 kb 5′ to *PICALM* on chromosome 11 (Harold et al., 2009; Lambert et al., 2013). *CLU* (rs1113600) is primarily involved with cholesterol metabolism, *CR1* (rs6656401) with immune response, and *PICALM* (rs3851179) with endocytosis (Karch and Goate, 2015). Approximately 50% of AD patients are *APOE* ε4+ carriers (Karch et al., 2014) and only 10–15% of AD risk is attributable to AD-related SNPs commonly identified in GWAS. This suggests that there is a large difference in risk associated with *APOE* versus SNPs identified in GWAS. For this reason, we chose to separately examine risk associated with *APOE* and the AD-GRS composed of *CLU*, *CR1*, and *PICALM.* All three SNPs have been associated with accelerated cognitive decline in older adults (Mengel-From et al., 2011; Liu et al., 2018; Zijlstra et al., 2018). These three SNPs (as polygenic risk scores or in network analyses) have been shown to interact with other genetic risk factors to predict cognitive trajectories (Darst et al., 2017; Sapkota and Dixon, 2018; Dixon and Lachman, 2019). To our knowledge, the present study is the first to examine all three SNPs and *APOE* in a risk score analysis with modifiable risk scores to predict cognitive trajectories in normal aging.

Recent studies have reported a range of modifiable risk factors (Zaninotto et al., 2018; McFall et al., 2019b; Peters et al., 2019) to increased risk of cognitive decline and dementia. Risk factors linked to AD risk in some recent studies include brain-gut-microbiota axis (Kowalski and Mulak, 2019), diabetes (Edwards et al., 2019), diet (Szczechowiak et al., 2019), stress and depression (Ross et al., 2018), traumatic brain injury (Ramos-Cejudo et al., 2018), and smoking (Niu et al., 2018). Key modifiable risk factors examined in the present study were PP (McFall et al., 2016), grip strength, and lifestyle activities, including social activities and cognitive-novel information processing (McFall et al., 2019b). All selected risk factors are AD-related but have also been shown to be associated with non-AD outcomes, such as differential cognitive decline in asymptomatic aging as well as other dementia disorders. A combined modifiable risk score with a greater number of risk indicators may have higher predictive power to detect EF performance and decline. We briefly review the component indicators of each of the risk domains, with emphasis on their prediction of cognitive change in asymptomatic aging as well as associations with dementia.

In the functional-health risk score, we include PP, grip strength, and BMI (McFall et al., 2019b). PP is a reliable proxy of arterial stiffness that shows linear increase across older adulthood and is considered a better predictor of poor vascular health compared to systolic or diastolic blood pressure (Raz et al., 2011; Nation et al., 2013; McFall et al., 2015; Sapkota et al., 2018). Higher PP has been associated with steeper decline in EF trajectories (McFall et al., 2015) and faster decline in global cognition with increasing age (Levine et al., 2019). Higher levels of PP associated with steeper EF decline is moderated by *APOE* and *CLU* allelic risk (McFall et al., 2016). Specifically, carriers of low allelic risk for *APOE* (ε2+) and *CLU* (T/T) showed less differential EF decline with higher PP levels than *APOE* (ε4+) and *CLU* (C/C) risk carriers. Grip strength has been associated with cognitive abilities such as verbal and spatial ability, processing speed, and memory in adults over the age of 65 years (Sternäng et al., 2016). Non-demented older adults with poor grip strength showed declining cognitive performance for EF, episodic memory, semantic memory, and crystallized ability (MacDonald et al., 2011). Inconsistent results have been observed for BMI risk: (1) high BMI was associated with higher dementia risk (Emmerzaal et al., 2015), (2) high mid-life BMI associated with changes in gray matter may translate into memory impairments (Kharabian Masouleh et al., 2016), (3) high BMI was associated with poorer EF and language performance (Schmeidler et al., 2019), and (4) high BMI was associated with less decline in EF, speed and memory domains selectively for females (Bohn et al., 2020).

In the lifestyle-reserve risk score, we include measures of physical activity, social activity, cognitive-integrative information processing, cognitive-novel information processing, and education (McFall et al., 2019b). Involvement in physically challenging activities have been shown to delay AD onset (Vemuri et al., 2012; Chen W.W. et al., 2016; Wang et al., 2019) and regular physical activity has been associated with lower incidence of cognitive impairment (Palta et al., 2019; Tomata et al., 2019). In non-demented aging, higher everyday physical activity has been associated with higher performance on EF (Thibeau et al., 2016), memory (Bherer et al., 2013), and visuospatial functions (Lin et al., 2019). Physical activity in middle life and late life may reduce the risk of neurodegenerative diseases by approximately 35–45% (Hamer and Chida, 2008). A review of longitudinal studies on social activity showed that poor or no social activity is linked to increased dementia risk (Wang et al., 2002) and more late life social activity is connected with reduced risk of incident dementia (Marioni et al., 2015). Overall, greater engagement in social activities throughout life was associated with less cognitive decline in later life (Wang et al., 2012). Cognitive activities involve mentally stimulating, novel, and integrative tasks such as puzzles or playing board games. Greater participation in cognitive activities has been linked to reduced dementia risk (Wang et al., 2012; Yates et al., 2016). We include education as a proxy marker for reserve (McFall et al., 2019b). High education has been associated with greater cognitive reserve and superior cognitive performance in older adults (Zahodne et al., 2011; Anstey et al., 2015; Livingston et al., 2017; Dixon and Lachman, 2019). Older adults with high education levels may also be more engaged in healthier lifestyle activities (i.e., physical activity) (Shaw and Spokane, 2008).

Previous risk score predictions have focused on dementia incidence as the primary outcome (Reitz et al., 2010; Sindi et al., 2015). We build and examine three modifiable risk scores to predict differential EF performance and further test how an AD genetic risk network (represented with *APOE* and AD-GRS) moderates all three associations in non-demented older adults. EF is among a selected set of cognitive domains (e.g., episodic memory and neurocognitive speed) that are prominently associated with important brain and cognitive changes in normal aging, with implications for emerging impairment and dementia (de Frias et al., 2006; Luszcz, 2011; Turner and Spreng, 2012; McFall et al., 2017; Reuter-Lorenz et al., 2021). EF is typically characterized as having three important components: mental set shifting, updating information, and inhibition of responses (Miyake et al., 2000; Miyake and Friedman, 2012; Schnitzspahn et al., 2013; Friedman and Miyake, 2017). All three EF dimensions have been shown to have important implications in brain and cognitive aging (Raz et al., 1998; Gunning-Dixon and Raz, 2003; Raz and Rodrigue, 2006; de Frias et al., 2009). Decline in EF performance has typically been observed in normal aging (Luszcz, 2011; Sapkota et al., 2015, 2017; Sapkota and Dixon, 2018; Reuter-Lorenz et al., 2021) and at the onset of cognitive impairment (de Frias et al., 2009; McFall et al., 2017) or AD (Bäckman et al., 2005; Grober et al., 2008). We examined two common markers of EF inhibition (Hayling Sentence Completion, and Stroop) and EF shifting (Brixton Spatial Anticipation and Color Trails) using a validated latent EF variable representation. Our EF latent variable represents the broader EF domain and has several important advantages to both single and composite scores. These include: (1) statistically superior and robust estimation of the EF construct by incorporating four standard and widely used EF tests and reducing the number of models tested, (2) measurement errors associated with each indicator are adjusted in the model, (3) longitudinal invariance is established for the EF latent variable across all three waves, and (4) the EF factor is generalizable and replicable when these four standard tests are examined and also with other similar and available tests (Little, 2013; Bohn et al., 2020).

### Research Questions

We examine *APOE* and the AD-GRS to test how genetic risk changes the association of modifiable risk scores on EF trajectories. The present study has three research questions (RQs).

#### Research Question 1

Do higher risk scores for (1) functional-health, (2) lifestyle-reserve, and (3) M-CRS, predict poorer EF performance and steeper decline? Which risk score has the highest predictive power to detect differences in EF performance and decline?

#### Research Question 2

Are the associations between the three modifiable risk scores and EF performance and decline moderated by *APOE* ε4+ risk?

#### Research Question 3

Is the *APOE* risk moderation for the association between three modifiable risk scores and EF further moderated by low and high AD-GRS?

### Expectations

First, we expect to observe that those with high risk score for functional-health, lifestyle-reserve, and M-CRS would have worse EF performance and steeper decline. The M-CRS will have the highest predictive power to detect EF changes. Second, this association between modifiable risk scores and EF would be moderated by *APOE* with poorer EF performance and steeper decline in the *APOE* ε4+ group. Third, the *APOE* moderation would be further moderated by AD-GRS with the worse EF performance and decline in the high AD-GRS. We further expect that these effects would be most evident with high M-CRS.

## Materials and Methods

### Participants

We used data from the Victoria Longitudinal Study (VLS), a large scale, longitudinal study examining biomedical, health, genetic, lifestyle, cognitive, and other aspects of aging and dementia. All volunteers in the VLS were recruited as cognitively healthy adults initially between 53 and 85 years. Volunteers were recruited through advertisements and received a small honorarium for their participation. Written informed consent was obtained from all participants. The VLS and all present data collection procedures are in full and certified compliance with prevailing human/institutional research ethics guidelines. All VLS participants are recruited relatively healthy, non-demented, no adverse health histories, especially those that would affect brain and cognition such as stroke or dementia. For the present study, we applied the following exclusionary criteria to the genotyped VLS sample: (1) anti-psychotic medication (*n* = 4), (2) Mini-Mental State Exam scores less than 24 (*n* = 1), (3) uncontrolled hypertension (*n* = 1), (4) insulin-controlled diabetes (*n* = 4), (5) history of serious head injury (e.g., hospitalized) (*n* = 8), and (6) *APOE* ε2/ε4 genotype (*n* = 30). Accordingly, we assembled an accelerated longitudinal design (spanning age 53–95 years) with 602 non-demented older adults [mean age = 70.63 (8.70) years; % female = 66]. The present sample uses three waves of data on participants differing in baseline age, thus creating a broad distribution of individualized cognitive trajectories (McFall et al., 2019b). In the present sample, a segment of the participants (across all ages) were not available for a third wave. The number of participants for each wave were: (1) Wave 1, *n* = 602, (2) Wave 2, *n* = 493, and (3) Wave 3, *n* = 277 (incomplete testing at time of study: *n* = 228). The average retention rates from Wave 1 to Wave 2 was 81% and Wave 2 to Wave 3 was 56%. However, taking into account the incomplete testing at the time of study (*n* = 228) for participants with a third-wave opportunity, the Wave 2 to Wave 3 retention rate was 88%. The average interval was 4.4 years between Wave 1 and Wave 2, and 4.5 years between Wave 2 and Wave 3.

### DNA Extraction and Genotyping

Saliva was collected according to standard procedures from Oragene DNA Genotek and stored at room temperature in Oragene^®^ disks until DNA extraction. Specific details on genotyping for *CLU* (rs11136000), *CR1* (rs6656401), *PICALM* (rs3851179), and *APOE* (rs7412, rs429358) are available elsewhere (Sapkota et al., 2017; Sapkota and Dixon, 2018). The genotype frequencies did not differ significantly from Hardy-Weinberg (HW) equilibrium for *APOE* (χ^2^ = 0.189, *p* = 0.66) and *CLU* (χ^2^ = 0.710, *p* = 0.40). We note that the *CR1* (χ^2^ = 6.219, *p* = 0.01) and *PICALM* (χ^2^ = 36.955, *p* = 0.00) genotype frequencies were not in HW equilibrium. Our sample comprises of similar number of homozygotes and heterozygotes for *CR1* (GG = 213, G/A = 332, A/A = 85) and *PICALM* (C/C = 151, C/T = 233, T/T = 245) which resulted in HW disequilibrium. We note that we are not using these two SNPs directly in our analysis but as contributing toward an overall genetic risk score. Deviation from HW equilibrium may be due to smaller sample size (in comparison to genome-wide association studies), population phenomena (i.e., natural selection) (Wishart et al., 2011), purifying selection, inbreeding, or copy number variation (Chen et al., 2017). HW calculations may be under powered or are not always reported in genetic association studies (Wittke-Thompson et al., 2005; Namipashaki et al., 2015). Deviations from HW with SNPs contributing toward an overall genetic risk score has been previously reported (Sapkota and Dixon, 2018). The *APOE* and AD-GRS groups from our previous work (Sapkota and Dixon, 2018) were used to examine moderation associations using *APOE* (risk = ε4+) and AD-GRS (*CLU* [risk = C+] + *CR1* [risk = A+] + *PICALM* [risk = T+]). For AD-GRS calculation, first, we dichotomized *CLU* (risk: C/C, C/T; no risk: T/T), *CR1* (risk: A/A, A/G; no risk: G/G), and *PICALM* (risk: T/T, T/C; no risk: C/C) into no risk (0) and risk (1) groups. Second, we summed across *CLU*, *CR1*, and *PICALM* to obtain a score for each adult ranging from 0 to 3. Third, we performed a median split for this score and grouped the *CLU* + *CR1* + *PICALM* allelic risk score by low (0–1 risk allele) and high (2–3 risk allele) genetic risk. *APOE* was grouped into no risk (ε4-) and risk (ε4+) groups. Participant characteristics by *APOE* genotype (ε4-/ ε4+) are displayed in Table 1, and as further stratified by low and high AD-GRS are displayed in Table 2.

**TABLE 1:** Baseline participant characteristics as stratified by *Apolipoprotein E* (*APOE*) genotype (ε4-/ε4+).

**Characteristics**	***APOE* ε4−**	***APOE* ε4+**
*n*	453	149
Age (years)	70.89 (8.34)	69.86 (8.27)
Education (years)	15.19 (2.97)	15.55 (3.07)
Sex (m/f)	149/304	56/93
Mini Mental State Exam	28.66 (1.24)	28.68 (1.25)
Physical activities	15.58 (5.19)	16.13 (5.15)
Social activities	22.76 (6.61)	21.85 (7.16)
Integrative information processing	18.73 (8.87)	20.20 (9.25)
Novel information processing	74.65 (17.27)	76.78 (15.63)
Pulse pressure (mm Hg)	51.56 (10.13)	51.61 (9.52)
Body mass index (kg/m^2^)	27.10 (4.38)	26.61 (3.74)
Grip strength (kg/f)	29.21 (9.53)	29.82 (9.01)
Diabetes (yes/no)	37/324	9/111
Depression (yes/no)	60/299	17/103
Hardening of arteries (yes/no)	45/315	12/108
Alcohol dependence (yes/no)	14/345	6/114

**TABLE 2:** Baseline participant characteristics as stratified by *Apolipoprotein E* (*APOE*) genotype (ε4-/ε4+) and Alzheimer’s disease genetic risk score (AD-GRS; *Clusterin* + *Complement receptor 1*+ *Phosphatidylinositol-binding clathrin assembly protein*).

**Characteristics**	***APOE* ε4−**		***APOE* ε4+**	
	**Low AD-GRS**	**High AD-GRS**	**Low AD-GRS**	**High AD-GRS**
*n*	79	371	17	132
Age (years)	69.12 (8.80)	71.29 (8.78)	71.37 (8.43)	69.66 (8.26)
Education (years)	15.44 (2.99)	15.15 (2.97)	14.76 (3.36)	15.65 (3.03)
Sex (m/f)	25/54	124/247	6/11	50/82
Mini Mental State Exam	28.87 (1.14)	28.63 (1.25)	28.24 (1.30)	28.73 (1.24)
Physical activities	16.29 (5.40)	15.40 (5.09)	15.00 (5.50)	16.28 (5.10)
Social activities	23.68 (6.18)	22.55 (6.68)	21.82 (5.09)	21.86 (7.40)
Integrative information processing	20.08 (8.57)	18.46 (8.93)	21.35 (9.66)	20.05 (9.22)
Novel information processing	77.79 (20.34)	70.07 (16.47)	75.76 (16.47)	76.91 (15.58)
Pulse pressure (mm Hg)	50.58 (10.45)	51.86 (10.03)	54.88 (7.90)	51.18 (9.66)
Body mass index (kg/m^2^)	26.81 (4.41)	27.18 (4.38)	26.45 (4.93)	26.63 (3.58)
Grip strength (kg/f)	28.60 (8.83)	29.39 (9.71)	28.52 (7.49)	29.99 (9.19)
Diabetes (yes/no)	5/55	32/266	1/15	8/96
Depression (yes/no)	8/51	52/245	5/11	12/92
Hardening of arteries (yes/no)	8/52	37/260	2/14	10/94
Alcohol dependence (yes/no)	1/58	13/284	0/16	6/98

### Executive Function Measures

Two dimensions of EF (inhibition and shifting) were each measured by two standard and frequently used tests for cognitive, clinical, and neurobiological studies in older adults (de Frias et al., 2006; McFall et al., 2014; Sapkota and Dixon, 2018). Specific details on the procedures followed and scoring for Hayling sentence completion (inhibition) (Burgess and Shallice, 1997), Stroop (inhibition) (Taylor et al., 1997), Brixton spatial anticipation (shifting) (Burgess and Shallice, 1997), and color trails (shifting) (D’elia et al., 1996) are available elsewhere (Bielak et al., 2006; Sapkota et al., 2015).

### Modifiable AD Risk Markers

*Functional-health* markers included baseline (1) PP [calculated with systolic blood pressure minus diastolic blood pressure (mmHg)], (2) grip strength [average hand strength (kg/force)], and (3) BMI [weight/height^2^ (kg/m^2^)].

*Lifestyle activities* were based on the VLS Activity Lifestyle Questionnaire (Small et al., 2012) used to determine the level or frequency of participation in everyday activities. For the present study we selected the following four domains: (1) social activity, such as visiting friends (7 items), (2) physical activity, such as gardening (4 items), (3) cognitive-integrative information processing, such as playing a musical instrument (12 items), and (4) cognitive-novel information processing, such as completing jigsaw puzzles (27 items). The frequency of participation is rated on a 9-point scale (never, less than once a year, about once a year, 2 or 3 times a year, about once a month, 2 or 3 times a month, about once a week, 2 or 3 times a week, daily).

*Reserve* was estimated by using education (total years) as a proxy (McFall et al., 2019b; Staekenborg et al., 2020).

### Statistical Analyses

We used structural equation modeling for all analyses with Mplus Version 7.4. The best fitting model was determined by examining several fit statistics. The chi-square test of model (χ^2^; *p* > 0.05) allowed for an overall indication of good model fit. Additional absolute/comparative fit indices were also examined to determine a good model fit to the data (Kline, 2010): the root mean square error of approximation (RMSEA ≤ 0.05), comparative fit index (CFI ≥ 0.95), and the standardized root mean square residual (SRMR ≤ 0.08). All missing values were assumed to be missing at random (attrition) or missing completely at random (item nonresponse) (Little, 2013). We used maximum likelihood (Enders, 2011, 2013) to estimate all EF factor scores due to attrition in Wave 2 (18%) and Wave 3 (26*%*). Any missing predictor values (0.5–13%) were removed from analysis. Although our study had relatively high retention rates (and thus relatively low drop-out rates) across the three waves of testing [Wave 1 to Wave 2 was 81%, Wave 2 to Wave 3 (after accounting for the incomplete testing at the time of study) was 88%], we followed the current best practices for missing data estimation with attention to accuracy and replicability. Specifically, for our missing at random data in a structural equation modeling framework, we used maximum likelihood estimation. This technique minimizes the risk of drawing incorrect conclusions due to inflated Type 1 and Type 2 error, biased parameter estimates, inflated confidence intervals, and loss of information resulting in reduced statistical power (Enders and Bandalos, 2001; Enders, 2005; Baker, 2019).

#### Modifiable Risk Score Calculations

Each risk indicator included in the functional-health and lifestyle-reserve risk scores was categorized and ranged from 0 to 2 (2 = greater risk; see Table 3) (Anstey et al., 2014; McFall et al., 2016; Sapkota et al., 2017).

**TABLE 3:** Weights assigned to calculate risk scores for functional-health and lifestyle-reserve domains.

	**Weights**
**Functional-health indicators**	
Pulse pressure (mm Hg)	
<52	0
52–72	1
>72	2
Grip strength (kg/f)	
Strong	0
Weak	1
Body mass index (kg/m^2^)	
Normal (18.5–25)	0
Underweight/overweight (< 8.5/25–30)	1
Obese (>30)	2
**Lifestyle-reserve indicators**	
Physical activities	
High	0
Low	1
Social activities	
High	0
Low	1
Cognitive-integrative information processing activities	
High	0
Low	1
Cognitive-novel information processing activities	
High	0
Low	1
Education (years)	
>11	0
8–11	1
<8	2

##### Functional-health risk score

Risk associated with three indicators was summed to obtain a total risk score ranging from 0 to 5. Specifically, PP was grouped into low (0) for adults with <52 mm Hg, moderate (1) for adults with PP between 52 and 72 mm Hg, and high (2) for adults with PP greater than 72 mm Hg. Grip strength was grouped into low (0) or high (1) using the mean (see Table 1) as a cut-off as stratified by *APOE* ε4-/ε4+ groups. BMI was grouped into normal (0) for adults with BMI from 18.5 to 25, underweight/overweight (1) for those with BMI < 18.5 and 25–30, respectively, and obese (2) for adults with BMI > 30.

##### Lifestyle-reserve risk score

Risk associated with the five indicators was summed to obtain a total risk score ranging from 0 to 6. Specifically, all lifestyle activities were grouped into lower and higher (Sapkota et al., 2017) where the mean (see Table 1) was used as the cut-off as stratified by *APOE* ε4-/ε4+ groups. Education was grouped into high (0) for adults with education greater than 11 years, moderate (1) for adults with 8–11 years of education, and low (2) for adults with less than 8 years of education.

##### Modifiable-composite risk score

The functional-health and lifestyle-reserve risk scores were summed to obtain a total risk score ranging from 0 to 11.

#### Foundational Statistical Analyses

##### Factor analyses for EF latent variable

We tested and confirmed a previously established one-factor EF latent variable. Specifically, confirmatory factor analysis (CFA) was used to examine loadings of all four manifest variables (Stroop, Hayling, Brixton, and Color trails) on the predicted latent variable. The first model tested all observed variables on one latent EF factor and the second model tested a two-factor shifting and inhibition model.

##### Longitudinal invariance

We established longitudinal invariance across all three waves for the best EF latent variable. First, we started with configural invariance, which establishes that all four indicators load on to the same factor. Second, metric invariance tests whether the unstandardized factor loadings at Waves 1–3 can be constrained and set to be equal to each other. Third, scalar invariance examines whether the four EF indicator intercepts can be constrained to be equal across all waves. Fourth, equal residuals invariance examines whether the EF factor can explain the same amount of variability across the three waves.

##### Latent growth model for EF

We determined the best latent growth model for the EF latent variable. We adopted a model building approach and started with a simple (null) model, and added parameters at each step to arrive at a baseline model of change. The null model assumes that there is no change over three waves, followed by the addition of fixed intercepts, random intercepts, fixed slope, random slope, fixed quadratic and random quadratic. First, in the null model, the variances for the intercepts were fixed across adults to 0. Second, in the random intercepts model, individuals were allowed to vary in intercept variance by removing the fixed intercept at 0. Third, a fixed linear slope was added to the baseline model by fixing the slope to 0 across all adults. The fixed linear slope assumed that all participants were changing in performance at the same rate. Fourth, adults were allowed to vary in their slope performance by removing the fixed linear slope constraint, and adding a random intercept and random linear slope model of change. Fifth, a fixed quadratic was added to the random intercept and random linear slope model, where both the intercepts and the slope were allowed to vary across individuals, but the curvilinear change was fixed across all participants. Following the examination of model fit, the χ^2^ difference statistic was calculated to detect any improvement in fit with the addition of free parameters at each step.

#### Main Statistical Analyses

Path analyses were conducted to examine associations of three modifiable risk scores on EF as moderated by AD genetic risk scores.

*RQ1.* EF was regressed on each modifiable risk score (functional-health, lifestyle-reserve, and M-CRS).

*RQ2.* To test *APOE* moderation, EF was regressed on all three risk scores as stratified by *APOE* ε4+ risk.

*RQ3.* To test subsequent moderation with AD-GRS, EF was regressed on all three risk scores as stratified by *APOE* ε4+ risk and subsequently stratified by low and high AD-GRS.

Due to sample size limitations with the two genetic moderation models, we did not test for sex differences in the present study. Instead, we included sex as a covariate in all models. Continuous age was used as the metric of change by transforming wave-based longitudinal design to age-based accelerated longitudinal design. We use chronological age (rather than, for example, wave) as the metric of change which takes into account all of the available ages (in years) that constitute the full age band of the study sample (i.e., 55–95 years). This 40-year band of aging fully accommodates each longitudinal trajectory produced by the individual participants, regardless of their starting age. The association between each independent variable and EF change is calculated and interpreted with age as essentially an integrated covariate. Statistically, this approach is better than indirectly using age through standard covariation techniques (Galbraith et al., 2014; McFall et al., 2016). Using age in this manner, we also take into account any EF changes due to age or risk factors directly influenced by age (such as *APOE* risk). To establish a baseline for our analyses, we centered chronological age at 75 years. Previous cognitive aging research has shown that age-related changes become evident at around 75 years (Small et al., 2011; Dixon et al., 2012), and this is consistent with recommended standards and other VLS research (Dixon et al., 2012; Thibeau et al., 2019; Bohn et al., 2020).

## Results

### Foundational Results

First, we established that the one-factor parsimonious model of EF provided the best fit to the data (Supplementary Table 1). Second, for longitudinal invariance, we obtained partial scalar longitudinal invariance for EF across all three waves (Supplementary Table 1). EF factor scores were computed and used in all subsequent models (see covariance coverage in Supplementary Table 2). Third, growth model analyses showed that the random intercept and random slope model was the best fit for the one-factor EF latent variable and was used in all subsequent analyses (Supplementary Table 3). To represent longitudinal data, EF factor scores were plotted in individualized trajectories over the 40-year band of aging (see Supplementary Figure 1). Taken together, these results were expected and showed that our one-factor EF construct best represents the overall characteristics of the four EF tests in our non-demented sample.

### Research Question Results

#### Research Question 1

All three modifiable risk scores significantly predicted EF performance and decline. Overall, higher risk scores predicted lower EF performance and steeper decline (see Table 4). This pattern of association applied to: (1) functional-health risk scores (level: β = −0.135, SE = 0.048, *p* = 0.005; slope: β = −0.007, SE = 0.002, *p* = 0.003), (2) lifestyle-reserve risk scores (level: β = −0.197, SE = 0.035, *p* < 0.001; slope: β = −0.009, SE = 0.002, *p* < 0.001), and (3) M-CRS (level: β = −0.161, SE = 0.031, *p* < 0.001; slope: β = −0.007, SE = 0.001, *p* < 0.001). Informally, the lifestyle-reserve risk score had the highest regression coefficient to predict EF performance and decline. Sex was a significant covariate in the analyses for all three modifiable risk scores, with men showing steeper EF decline.

**TABLE 4:** Unstandardized regression coefficients and model fit indices by research question for all models.

	**Intercept**			**Slope**			**Model fit indices**				
	**β**	**SE**	***p***	**β**	**SE**	***p***	**H0 value**	**Free parameters**	**−2LL**	**AIC**	**BIC**
**Research Question 1**											

FRS (*n* = 524)	–0.135	0.048	0.005	–0.007	0.002	0.003	–700.892	12	1,401.784	1,425.784	1,476.922
Sex	0.203	0.106	0.055	0.013	0.005	0.008					
LRS (*n* = 595)	–0.197	0.035	0.000	–0.009	0.002	0.000	–805.219	12	1,610.438	1,634.439	1,687.102
Sex	0.146	0.092	0.112	0.009	0.004	0.047					
M-CRS (*n* = 520)	–0.161	0.031	0.000	–0.007	0.001	0.001	–687.957	12	1,363.914	1,387.914	1,438.960
Sex	0.275	0.103	0.008	0.015	0.005	0.001					

**Research Question 2**											

***APOE* ε4−**											
FRS (*n* = 391)	–0.140	0.052	0.007	–0.007	0.002	0.002	–689.055	24	1,378.110	1,426.110	1,528.386
Sex	0.273	0.123	0.026	0.017	0.005	0.002					
LRS (*n* = 447)	–0.210	0.039	0.000	–0.009	0.002	0.000	–790.016	24	1,580.032	1,628.031	1,733.357
Sex	0.217	0.101	0.032	0.012	0.005	0.014					
M-CRS (*n* = 387)	–0.168	0.033	0.000	–0.007	0.002	0.000	–668.758	24	1,337.516	1,385.515	1,487.607
Sex	0.357	0.114	0.002	0.020	0.005	0.000					
***APOE* ε4+**											
FRS (*n* = 133)	–0.149	0.124	0.230	–0.006	0.006	0.309	–689.055	24	1,378.110	1,426.110	1,528.386
Sex	–0.021	0.255	0.935	0.001	0.012	0.940					
LRS (*n* = 148)	–0.165	0.083	0.048	–0.008	0.004	0.035	–790.016	24	1,580.032	1,628.031	1,733.357
Sex	–0.083	0.220	0.707	–0.001	0.010	0.902					
M-CRS (*n* = 133)	–0.157	0.080	0.048	–0.007	0.004	0.053	–668.758	24	1,337.516	1,385.515	1,487.607
Sex	0.024	0.256	0.925	0.003	0.012	0.797					

**Research Question 3**											

**Low AD-GRS in *APOE* ε4− group**											
FRS (*n* = 66)	0.089	0.135	0.510	0.001	0.005	0.796	–460.739	24	921.478	969.479	1,064.543
Sex	–0.035	0.350	0.920	0.009	0.014	0.533					
LRS (*n* = 78)	–0.269	0.108	0.013	–0.015	0.005	0.006	–543.272	24	1,086.544	1,134.544	1,232.844
Sex	–0.080	0.304	0.792	–0.001	0.013	0.950					
M-CRS (*n* = 66)	–0.110	0.088	0.209	–0.006	0.004	0.082	–443.038	24	886.076	934.077	1,028.892
Sex	0.169	0.333	0.612	0.014	0.013	0.285					
**High AD-GRS in *APOE* ε4− group**											
FRS (*n* = 322)	–0.204	0.056	0.000	–0.010	0.003	0.000	–460.739	24	921.478	969.479	1,064.543
Sex	0.333	0.130	0.010	0.018	0.006	0.004					
LRS (*n* = 366)	–0.191	0.043	0.000	–0.007	0.002	0.002	–543.272	24	1,086.544	1,134.544	1,232.844
Sex	0.274	0.112	0.015	0.014	0.006	0.014					
M-CRS (*n* = 318)	–0.179	0.036	0.000	–0.007	0.002	0.000	–443.038	24	886.076	934.077	1,028.892
Sex	0.391	0.123	0.000	0.019	0.006	0.002					
**Low AD-GRS in *APOE* ε4+ group**											
FRS (*n* = 15)	–0.437	0.397	0.270	–0.011	0.035	0.761	–197.268	20	394.536	434.536	492.343
Sex	Not a good model fit										
LRS (*n* = 17)	–0.317	0.497	0.523	–0.016	0.018	0.370	–221.547	24	443.094	491.094	563.027
Sex	–0.915	1.022	0.371	–0.022	0.052	0.677					
M-CRS (*n* = 15)	–0.341	0.224	0.146	–0.005	0.016	0.775	–188.715	24	377.430	425.429	494.798
Sex	–0.771	0.773	0.318	–0.042	0.036	0.245					
**High AD-GRS in *APOE* ε4+ group**											
FRS (*n* = 118)	–0.092	0.126	0.466	–0.005	0.006	0.335	–197.268	20	394.536	434.536	492.343
Sex			Not a good model fit								
LRS (*n* = 131)	–0.164	0.094	0.080	–0.008	0.004	0.066	–221.547	24	443.094	491.094	563.027
Sex	0.022	0.242	0.927	0.002	0.012	0.884					
M-CRS (*n* = 118)	–0.127	0.089	0.154	–0.006	0.004	0.110	–188.715	24	377.430	425.429	494.798
Sex	0.130	0.284	0.648	0.007	0.014	0.616					

*SE = standard error; H0 = log likelihood; −2LL = −2 log likelihood; AIC = Akaike Information Criteria; BIC = Bayesian Information Criteria. FRS = functional-health risk score; LRS = lifestyle-reserve risk score; M-CRS = Modifiable-Composite Risk Score (FRS + LRS). Sex was included as a covariate in all the models.*

#### Research Question 2

The *APOE* moderation results are presented separately for each modifiable risk score. First, we observed that higher functional-health risk scores predicted poorer EF performance and steeper decline (level: β = −0.140, SE = 0.052, *p* = 0.007; slope: β = −0.007, SE = 0.002, *p* = 0.002) selectively for the *APOE* ε4− group (Table 4). Second, *APOE* ε4+ risk did not moderate the prediction of lifestyle-reserve risk scores (stronger predictor) on EF performance and change. Third, higher M-CRS predicted steeper EF decline (slope: β = −0.007, SE = 0.033, *p* < 0.001) selectively for the *APOE* ε4− group. Sex was a significant covariate in the analyses for both significant moderations in the *APOE* ε4− group; specifically, men had lower EF performance and steeper decline than women (see Table 4).

#### Research Question 3

**FIGURE 2:**
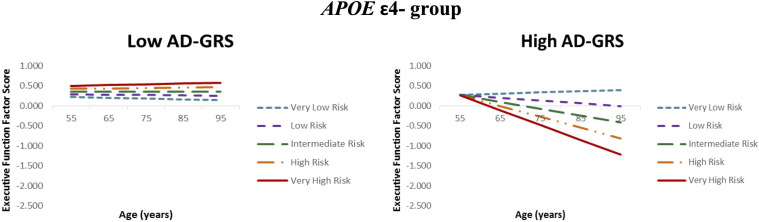
Executive function trajectories for functional-health risk score as moderated by AD genetic risk. *APOE* ε4 non-carriers with increasing functional-health risk scores had poorer EF performance and steeper 40-year trajectories selectively in the high AD-GRS group. Functional-health risk score is coded from very low risk (dashed blue line) to very high risk (solid red line).

Given the previous results (RQ2), we focus the AD-GRS analyses on the *APOE* ε4− group. First, higher functional-health risk scores predicted poorer EF performance and steeper decline (level: β = −0.204, SE = 0.056, *p* < 0.001; slope: β = −0.010, SE = 0.003, *p* < 0.001) selectively in the high AD-GRS group (Figure 2). Second, AD-GRS did not moderate the prediction of lifestyle-reserve risk scores on EF performance and change. Third, higher M-CRS predicted poorer EF performance and steeper decline (level: β = −0.179, SE = 0.036, *p* < 0.001; slope: β = −0.007, SE = 0.002, *p* < 0.001) selectively in the high AD-GRS group. Sex was a significant covariate in the analyses for both moderations in *APOE* ε4− group for adults with high AD-GRS (see Table 4), where men had lower EF performance and steeper decline. AD-GRS did not moderate the prediction of modifiable risk scores on EF performance and change (Table 4) in the *APOE* ε4+ group.

## Discussion

We examined a series of interactive associations using modifiable and genetic risk scores to predict EF trajectories in a longitudinal study of non-demented older adults. Our goals were to (1) test and compare two sets of modifiable risk scores (and their combination) to determine relative predictive power for EF trajectories, (2) identify whether the key AD genetic risk factor (*APOE*) moderates the expected associations between the modifiable risk scores and EF change, and (3) determine whether an AD-GRS influences the moderation between *APOE* and modifiable risk scores in predicting EF. Overall, the results were consistent with the expectation that in non-demented aging, differential cognitive trajectories were predicted and moderated by a network of AD-related risk factors that include two forms of both modifiable indicators (functional-health and lifestyle-reserve) and AD genetic risk markers [penetrant (*APOE*) and multi-genetic (AD-GRS)]. Such interactive and network approaches to biomarker predictions of asymptomatic brain and cognitive aging have appeared in several recent reports (Sapkota et al., 2017; Sapkota and Dixon, 2018; Licher et al., 2019; Lourida et al., 2019). The present study extends these reports in that it incorporates, sequentially, functional-health, lifestyle-reserve, and non-modifiable genetic modalities of AD risk.

Past studies have mainly focused on combining a wide range of modifiable risk indicators and genetic risk factors to create an overall risk score (Stephen et al., 2017; Licher et al., 2018) or examined PGRS using a large number of genetic risk variants (Andrews et al., 2019; Bakulski et al., 2020). Specific novelties of the present study include: (1) a broad representation of the functional-health risk domain, with contributions from three markers often used independently in candidate biomarker studies (i.e., PP, grip strength, BMI) (Walters et al., 2016), (2) an overall representation of the lifestyle-reserve domain, with contributions from five markers often examined in dementia and cognitive resilience studies (i.e., physical activity, social activity, novel activity, integrative activity, education), (3) examining the commonly studied genetic risk (*APOE*) in addition to a genetic risk score (AD-GRS) (Stephen et al., 2017), and (4) testing interactive associations between three modifiable risk scores with two genetic score moderations on EF trajectories.

Although the central finding of this study is that a network of interactions among modifiable and non-modifiable risk biomarkers operate to moderate asymptomatic cognitive trajectories, we now briefly unpack each of the components in the sequence of analyses. For RQ1, higher modifiable risk scores for the two domains (functional-health and lifestyle-reserve) and their combination (M-CRS) predicted poorer EF performance and steeper decline. Consistent with previous studies, we observed that risk indicators in all three risk scores significantly contributed toward poorer cognitive performance and accelerated decline (Hughes et al., 2009; Gunstad et al., 2010; Marioni et al., 2012, 2014; McFall et al., 2014; McDade et al., 2016; Albanese et al., 2017; Viscogliosi et al., 2017; Kivipelto et al., 2018; Kobayashi-Cuya et al., 2018; Bohannon, 2019; Bowman et al., 2019; Clouston et al., 2019; Kim et al., 2019). Although we expected to observe that the M-CRS would have the highest predictive power, we observed that the lifestyle-reserve risk score had the highest predictive power (level: β = −0.197; slope: −0.009) for EF performance and decline. Although the M-CRS provides an overall risk score, our results suggest that such risk scores should be constructed and validated with close attention to the fit and incremental value of each indicator. Including risk indicators in an overall risk score does not necessarily optimize the predictive performance of the composite measure. A precision approach to the building of composite risk scores is recommended, just as it is tested at the level of multi-modal networks. Our novel lifestyle-reserve risk domain with five indicators (physical activities, social activities, cognitive-integrative activities, cognitive-novel activities, education) selectively showed higher predictive power for EF trajectories (1) than the combined multi-component risk score and (2) regardless of the AD genetic risk contribution in non-demented older adults. We note three aspects of novelty in this finding. First, we are not aware of other studies using a combination of similar risk indicators to build a lifestyle-reserve risk score and subsequently predict EF trajectories in non-demented aging. Second, this is the first study to provide a comparison of the predictive power between lifestyle-reserve risk score and a combined-modifiable risk score composite with the lifestyle-reserve risk score showing higher predictive power for EF performance and decline. Third, our findings imply that elevated lifestyle-reserve risk scores are associated with EF decline in non-demented older adults regardless of AD genetic risk contribution. Future research in this area may benefit from compiling multiple relevant risk indicators to build domain-related risk scores and systematically testing each risk domain independently and in interaction with other domains of risk (including genetic risk) to predict differential cognitive trajectories in older adults.

For RQ2, *APOE* ε4+ risk moderated the association between modifiable risk scores and EF trajectories, whereby adults in the *APOE* ε4− group had poorer EF performance and steeper decline with higher M-CRS. A previous VLS study reported a similar pattern, whereby *APOE* moderation for lifestyle activities (integrative and novel information processing activities) was associated with cognitive decline only in *APOE* ε4 non-carriers (Runge et al., 2014). A recent report (Licher et al., 2019) using older adults from the Rotterdam Study, showed that low modifiable risk may only be associated with lower dementia risk in those with low and intermediate genetic risk. These protective associations were not observed in *APOE* ε4+ risk carriers. *APOE* risk carriers may already be at a disadvantage for poorer EF performance and steeper decline (i.e., a floor effect). Therefore, modifiable risk scores may not a play a significant role in cognitive change for *APOE* ε4+ carriers who are declining regardless of health and lifestyle factors. In addition, adults with less genetic risk such as *APOE* ε2 homozygotes may be more inclined to participate in higher lifestyle activities leading to added protection from key modifiable risk markers (Ngandu et al., 2015; Peters et al., 2019). Previous studies have also shown that *APOE* ε2+ carriers have slower EF decline (Reas et al., 2019), better EF and episodic memory performance (Sinclair et al., 2017), and less memory decline in the presence of poor vascular health (represented with PP) selectively for women *APOE* ε2+ carriers (McFall et al., 2019a). Regarding potential underlying mechanisms, *APOE* ε2+ carriers may show protective effects by using compensatory mechanisms; specifically, recent study showed that amnestic mild cognitive impairment adults had increased functional connectivity in the entorhinal cortex network for *APOE* ε2+ carriers (Chen J. et al., 2016). In the present study, *APOE* did not moderate the association between lifestyle-reserve risk scores and EF suggesting that older adults with high lifestyle risk scores may be at higher risk for EF decline regardless of their genetic risk status. In addition to *APOE* ε4+ risk stratification, future studies should also consider examining *APOE* protection as stratified by ε2− versus ε2+.

As expected, we observed that the M-CRS had a higher risk coefficient (β = −0.160) for EF performance than the functional health risk score (β = −0.148) in the *APOE* ε4− group. Higher risk prediction with the M-CRS suggests that functional-health risk factors alone may not be enough to observe a precise and accurate genetic moderation. Future studies should consider that a limited number of modifiable risk indicators (only functional-health or only lifestyle-reserve) may not provide a complete picture of the dynamic risk processes involved in cognitive changes as moderated by key neurocognitive genotypes. A multimodal network approach accounting for both modifiable and genetic risk may be required to detect accurate changes in cognitive trajectories and this may also vary by cognitive domain.

For RQ3, as expected, AD-GRS further moderated the association between modifiable risk scores and EF trajectories in the *APOE* ε4− group. Specifically, adults in the high AD-GRS group had poorer EF performance with increasing M-CRS. This suggests that only those with high AD-GRS combination were particularly vulnerable in our risk score network (see Figure 2) and emphasizes the importance of accounting for key AD genetic risk factors in addition to *APOE*. The M-CRS as moderated by AD genetic risk had higher predictive coefficient (β = −0.179) for EF performance than M-CRS alone (β = −0.168). Our multifactorial network showed that modifiable risk scores are moderated by an AD genetic risk (*APOE* and AD-GRS) on EF predictions. As part of post-hoc analyses, we observed that *APOE* ε2+ carriers (versus *APOE* ε2-) were potentially protected from the deleterious effects of increasing modifiable risk in this group. However, we do note that we had unequal sample sizes in the two groups (*n* = 62 in the ε2+ group and *n* = 309 in the ε2− group). The present approach advances previously studied risk scores in several ways. First, we distinguish between three modifiable risk scores and two genetic risk scores to examine a network of risk scores versus previous work focusing on an overall risk score (Anstey et al., 2014) or testing interactions between lifestyle risk and cognitive aging genetic risk score (Sapkota et al., 2017). Second, we test three modifiable risk scores to determine the risk score with the most predictive power. Previous studies have not differentiated between different modifiable risk domains to test domain-specific modifiable risk scores versus an overall risk score (Deckers et al., 2018). Third, we test genetic moderation for all three modifiable risk scores with *APOE* risk stratification followed by a previously published AD-GRS (Sapkota and Dixon, 2018) to predict EF trajectories in non-demented older adults. Future studies may benefit from applying similar multimodal network approach using modifiable and non-modifiable risk scores to predict cognitive trajectories in older adult populations.

Sex differences in cognitive aging risk profiles have been consistently observed (McDermott et al., 2016; Gurvich et al., 2018; McFall et al., 2019b) and commonly used as a risk indicator in risk scores (Anstey et al., 2014). Although we covaried for sex in all our models, we did not examine for sex differences in the present study due to our sample size limitations. As a significant covariate in our significant models (see Table 4), we observed that men showed steeper EF performance and/or decline than women. Our findings are consistent with recent VLS work where higher BMI was associated with less decline for women (Bohn et al., 2020), and higher number of memory resilience predictors were observed in women (McDermott et al., 2016). Future studies focusing on cognitive aging and dementia modifiable risk scores should consider interactive effects of complex genetic risk scores as stratified by sex to detect distinct differences in asymptomatic aging profiles.

We now mention several strengths and limitations of the present study. A first strength is a relatively large sample of older adults (*n* = 602) examined at three waves across a 40-year band of aging (age range = 53–95 years old). Our sample size allowed us to detect genetic moderations of modifiable risk scores on EF trajectories. Future studies with larger sample sizes may benefit by expanding our network approach to include additional AD genetic risk factors and modifiable risk domains to test a larger intricate network of risk scores on cognitive trajectories in both older adults and dementia populations. Second, our EF latent variable included four standard cognitive tests. We represent a broad construct domain that reduces measurement error associated with single EF tests. Third, we applied a longitudinal design with age as the metric of change. This approach incorporates chronological age directly into our analyses (Sapkota et al., 2017) and accounts for age-associated variability which is better than using age as a covariate in our statistical model. Fourth, our latent growth modeling approach in Mplus also accounts for missing data by using maximum likelihood estimation to generate factor scores for the dependent variable. Fifth, we extended previous cross-sectional and longitudinal studies (Sapkota et al., 2015, 2017; Sapkota and Dixon, 2018) testing synergistic associations of genetic risk factors alone to include the association of modifiable risk score on EF as moderated by AD genetic risk.

For limitations, first, our M-CRS included risk scores from only two domains (functional-health domain and lifestyle-reserve). Future work should consider including other risk indicators from various domains such as diabetes, stroke, depression, and smoking (Rochoy et al., 2019) among other identified key AD risk factors (Livingston et al., 2017). In addition, studies extending our multimodal network approach should consider an even broader range of AD-related risk factors, including clinical biomarkers (molecular, imaging) of AD diagnosis and progression, as well as related disorders. AD-specific molecular biomarkers are not routinely collected in asymptomatic aging studies and were not available in the current database. Second, we focused on EF trajectories, and future studies should consider including other cognitive domains (i.e., episodic memory) to examine genetic risk moderation between modifiable risk scores and cognitive trajectories. We also note that some of the risk factors in our network may apply to other disease conditions (i.e., diabetes) and may be used to predict cognitive trajectories in diabetes or other neurodegenerative conditions. Third, as we were continuing with the same group of participants from previous study with established AD-GRS (Sapkota and Dixon, 2018), the longitudinal design did not include a third wave for all participants. However, our results were not compromised because we used all data points available for each participant and confirmed that the latent EF variable was measurement invariant [partial scalar invariance level; (Kline, 2010)]. Fourth, although we co-varied for sex in our analyses, future work with larger sample sizes should consider stratifying by sex and then testing for differences in the extent to which complex multifactorial networks predict differential cognitive trajectories in older adults (Tierney et al., 2017). Fifth, we believe that low power did not play a role in our structural equation models with *n* > 100 (Little, 2013) in both *APOE* ε4− and ε4+ groups for two main reasons. We observed that for increasing lifestyle-reserve risk scores, both ε4− and ε4+ groups showed significant decline suggesting that power may not be an issue in the lower sample size group. In our previous genetic risk score study using the same sample (Sapkota and Dixon, 2018), we showed that high cognitive aging genetic risk score significantly predicted poorer EF performance and steeper decline in the *APOE* ε4+ group (lower sample size group). However, we note that future studies should consider using a larger sample size for similar models, a larger range of modifiable risk indicators to build a risk score, and different AD genetic polymorphisms that have been associated with *APOE* genotype (i.e., *TOMM40*; Lyall et al., 2014). Sixth, we observed HW deviations for two of our SNPs (*CR1* and *PICALM*) contributing to the overall AD-GRS. We observed reasonable and predictable results with the AD-GRS, which has previously been examined with EF trajectories (Sapkota and Dixon, 2018). Future studies should consider using a larger sample size with a range of neurodegenerative patients to test whether our non-demented sample played an important role in the HW deviations observed for *CR1* and *PICALM.* Seventh, we note that our participants were predominantly White, not of Hispanic origin, and that genotype allelic frequencies may vary in different population groups. Future studies should consider using adults diagnosed with neurodegenerative disorders and participants from other racial backgrounds to establish generalizability. Eighth, all VLS participants are initially screened for clinically diagnosed neurodegenerative conditions prior to enrollment and at each wave of testing. In addition to this screening, we applied additional exclusionary criteria (anti-psychotic medication, Mini-Mental State Exam scores less than 24, uncontrolled hypertension, insulin-controlled diabetes, history of serious head injury, *APOE* ε2/ε4 genotype) to ensure that all participants were normally aging throughout the longitudinal data collection. However, with no subsequent assessments (after the conclusion of this study), we cannot confirm that some participants would not transition later into preclinical phases. One participant in our sample was reported to have a dementia diagnosis at death and their EF data was not included at wave 3. Future work should consider deploying subsequent assessments and retroactively examining potential differential patterns for those who continued as asymptomatic and those who later developed cognitive impairment or dementia.

In conclusion, a multimodal network of risk factors predicted EF trajectories in non-demented older adults. To our knowledge, this is the first study to detect a genetic moderation (using *APOE* and AD-GRS) with modifiable risk scores (risk indicators: BMI, PP, grip strength, daily lifestyle activities, and education) on EF performance and change. Our key novel findings include: (1) lifestyle-reserve risk score alone had the highest predictive power for EF decline, (2) *APOE* moderated the association between M-CRS and EF performance and decline where only adults in the *APOE* ε4− group were significantly influenced by higher modifiable risk scores, (3) further AD-GRS genetic stratification in *APOE* ε4− provided a more precise illustration of older adults with the highest risk profile for EF decline. Examining multimodal associations using a network approach (including additive risk scores, moderation, and effect modification) may add an extra layer of precision to single risk score studies to detect older adults with high cognitive decline risk profiles, and also provide important insight for future intervention trials aimed at modifiable risk factors and neurocognitive aging.

## Data Availability Statement

The datasets presented in this study can be found in online repositories. The names of the repository/repositories and accession number(s) can be found below: https://sites.ualberta. ca/∼vlslab/index.html. We will make the data available to qualified researchers on a project website.

## Ethics Statement

The studies involving human participants were reviewed and approved by the Human Research Ethics Guidelines, University of Alberta. The patients/participants provided their written informed consent to participate in this study.

## Author Contributions

SS and RD designed the study and planned the analytic approach. SS performed the statistical analyses and wrote the first draft. All authors contributed to writing, editing, and interpretation.

## Conflict of Interest

The authors declare that the research was conducted in the absence of any commercial or financial relationships that could be construed as a potential conflict of interest.

## Publisher’s Note

All claims expressed in this article are solely those of the authors and do not necessarily represent those of their affiliated organizations, or those of the publisher, the editors and the reviewers. Any product that may be evaluated in this article, or claim that may be made by its manufacturer, is not guaranteed or endorsed by the publisher.
