# Supplementary material for: A Multimodal Risk Network Predicts Executive Function Trajectories in Non-demented Aging
Source: Front Aging Neurosci. 2021 Sep 16;13:621023. doi: 10.3389/fnagi.2021.621023 (PMC8482841; doi:10.3389/fnagi.2021.621023)

**Supplementary Tables and Figure**

Supplementary Table 1.

*Confirmatory factor analysis and longitudinal invariance model fit statistics and chi-square difference test for executive function factor for waves 1-3.*

|  | **AIC** | **BIC** | $\boldsymbol{\chi}_{\boldsymbol{M}}^{\boldsymbol{2}}$**(**$\boldsymbol{df}_{\boldsymbol{M}}$**)** | **RMSEA (90% CI)** | **CFI** | **SRMR** | $\boldsymbol{\chi}_{\boldsymbol{D}}^{\boldsymbol{2}}$ **(**$\boldsymbol{df}_{\boldsymbol{D}}$**)** |
| --- | --- | --- | --- | --- | --- | --- | --- |
| **Confirmatory Factor Analysis** | | | | | | | |
| W1 | 12263.880 | 12417.267 | 3.011 (2); *p* = 0.222 | 0.028 (0.00-0.089) | 0.993 | 0.015 | -- |
| W2 | 10624.309 | 10675.332 | 0.239 (2); *p* = 0.887 | 0.000 (0.000-0.041) | 1.000 | 0.004 | -- |
| W3 | 5738.166 | 5782.450 | 2.901 (2); *p* = 0.235 | 0.039 (0.000-0.129) | 0.991 | 0.021 | -- |
| **Longitudinal Invariance** | | | | | | | |
| Configural | 27666.008 | 27884.004 | 65.528 (41); *p* = 0.009 | 0.031 (0.016-0.044) | 0.984 | 0.077 | -- |
| Metric | 27661.656 | 27852.958 | 73.176 (47); *p* = 0.009 | 0.030 (0.015-0.042) | 0.983 | 0.079 | 7.648 (6) |
| Scalar | 27775.311 | 27939.920 | 198.832 (53); *p* = 0.000 | 0.066 (0.056-0.076) | 0.906 | 0.104 | 125.656 (6)** |
| Partial scalar^a^ | 27669.082 | 27851.487 | 84.602 (49); *p* = 0.001 | 0.034 (0.021-0.046) | 0.977 | 0.084 | 11.426 (2)* |

*Note.* AIC = Akaike Information Criteria; BIC = Bayesian Information Criteria; $\chi_{M}^{2}$ *=* Chi-square test of model fit; ${df}_{M}$ = Degrees of freedom for model fit; RMSEA = Root Mean Square Error of Approximation; CI = Confidence Interval; CFI = Comparative Fit Index; SRMR = Standardized Root Mean Square Residual; $X_{D}^{2}$ = Chi-square test of difference; ${df}_{D}$ = Degrees of freedom for difference in model fit; W = Wave.

**p*<.05; ***p*<.001.

^a^Partial scalar, where the intercept for Hayling and Stroop were constrained to be equal across all three time points.

Supplementary Table 2.

*Covariance coverage of four standard neuropsychological tests used to represent the executive function factor across three waves.*

|  | **Brixton W1** | **Brixton W2** | **Brixton W3** | **Color Trails W1** | **Color Trails W2** | **Color Trails W3** | **Hayling W1** | **Hayling W2** | **Hayling W3** | **Stroop W1** | **Stroop W2** | **Stroop W3** |
| --- | --- | --- | --- | --- | --- | --- | --- | --- | --- | --- | --- | --- |
| **Brixton W1** | 0.984 |  |  |  |  |  |  |  |  |  |  |  |
| **Brixton W2** | 0.802 | 0.812 |  |  |  |  |  |  |  |  |  |  |
| **Brixton W3** | 0.451 | 0.434 | 0.457 |  |  |  |  |  |  |  |  |  |
| **Color Trails W1** | 0.978 | 0.807 | 0.456 | 0.994 |  |  |  |  |  |  |  |  |
| **Color Trails W2** | 0.805 | 0.805 | 0.434 | 0.810 | 0.815 |  |  |  |  |  |  |  |
| **Color Trails W3** | 0.449 | 0.432 | 0.456 | 0.454 | 0.432 | 0.456 |  |  |  |  |  |  |
| **Hayling W1** | 0.970 | 0.797 | 0.445 | 0.972 | 0.801 | 0.443 | 0.978 |  |  |  |  |  |
| **Hayling W2** | 0.810 | 0.810 | 0.435 | 0.815 | 0.813 | 0.434 | 0.805 | 0.820 |  |  |  |  |
| **Hayling W3** | 0.462 | 0.441 | 0.457 | 0.467 | 0.441 | 0.456 | 0.456 | 0.443 | 0.468 |  |  |  |
| **Stroop W1** | 0.979 | 0.807 | 0.453 | 0.991 | 0.810 | 0.451 | 0.973 | 0.815 | 0.464 | 0.995 |  |  |
| **Stroop W2** | 0.805 | 0.805 | 0.434 | 0.810 | 0.810 | 0.432 | 0.801 | 0.813 | 0.441 | 0.812 | 0.815 |  |
| **Stroop W3** | 0.449 | 0.432 | 0.456 | 0.454 | 0.432 | 0.454 | 0.443 | 0.434 | 0.456 | 0.453 | 0.434 | 0.456 |

*Note.* W = Wave. Brixton = Brixton Spatial Anticipation Test. Hayling = Hayling Sentence Completion Test.

Supplementary Table 3.

*Latent growth model fit statistics and chi-square difference test for executive function by age.*

| **Model** | **H0 value** | **Free Parameters** | **-2LL** | **AIC** | **BIC** | ***D* (**$\boldsymbol{df}_{\boldsymbol{D}}$**)** |
| --- | --- | --- | --- | --- | --- | --- |
| Fixed Intercept | -2067.192 | 4 | 4134.384 | 4142.384 | 4160.180 | -- |
| Random Intercept | -1248.233 | 5 | 2496.466 | 2506.467 | 2528.711 | 1637.918 (1) |
| Random Intercept, Fixed Slope | -1229.368 | 6 | 2458.736 | 2470.736 | 2497.429 | 37.73 (1)** |
| Random Intercept, Random Slope | -868.207 | 8 | 1736.414 | 1752.413 | 1788.004 | 722.322 (2)** |
| Random Intercept, Random Slope, Fixed Quadratic | 1263.880 | 9 | 2527.760 | 2545.760 | 2585.800 | -791.346 (1) |

*Note.* H0 = Log Likelihood; -2LL = -2 Log Likelihood; AIC = Akaike Information Criteria; BIC = Bayesian Information Criteria; *D* = Deviance statistic; ${df}_{D}$ = Degrees of freedom for difference in deviance statistics.

**p*<.05; ***p*<.001.

*Supplementary Figure 1.* Individual trajectories of executive function €F factor scores over 40-year band of aging.


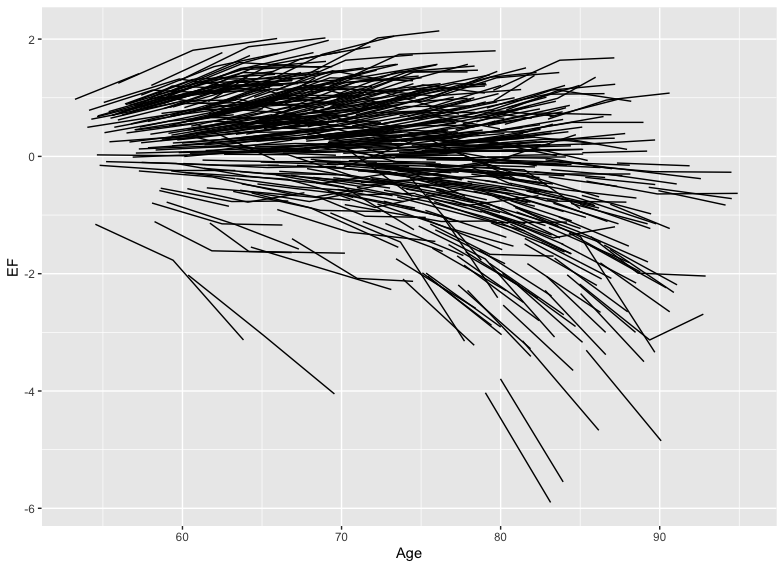

Supplement: Supplementary file 1 [file Data_Sheet_1.docx]
